# Prevalence, associated factors and medication for symptoms related to gastroesophageal reflux disease among 1114 private-tuition students of Anuradhapura, Sri Lanka

**DOI:** 10.1186/s12876-020-01193-3

**Published:** 2020-02-27

**Authors:** Darsha Gunasinghe, Chathurika Gunawardhana, Shakthi Halahakoon, Ali Haneeka, Najiyya Hanim, Chamara Hapuarachchi, Devarajan Rathish

**Affiliations:** 1 Faculty of Medicine and Allied Sciences, Rajarata University of Sri Lanka, Saliyapura, Anuradhapura, Sri Lanka; 2 Department of Pharmacology, Faculty of Medicine and Allied Sciences, Rajarata University of Sri Lanka, Saliyapura, Anuradhapura, Sri Lanka

**Keywords:** School children, Boarded, Midnight snacks, Lack of breakfast, Quick eating, Inadequate sleep

## Abstract

**Background:**

Gastroesophageal reflux disease (GORD) is a chronic and a common condition worldwide which causes mild to severe symptoms. Private tuition attendees are a group which could have potential risk factors for GORD. Therefore, we aimed to determine the prevalence, associated factors and medication for symptoms related to GORD among advanced level private-tuition attendees of Anuradhapura, Sri Lanka.

**Methods:**

A descriptive cross-sectional study was conducted among students aged ≥18 years. A self-administered questionnaire was used to collect data. Students scoring ≥ eight on the Frequency Scale for Symptoms of GORD were categorised to have symptoms related to GORD. Logistic regression was performed to determine the significant association between the variables of interest and the presence of symptoms related to GORD (*P* < 0.05).

**Results:**

Data of 1114 students were included for the analysis. A high prevalence of symptoms related to GORD (52% - 580/1114) was noted. Heartburn received the highest score among GORD symptoms. Biology students had the highest prevalence of GORD symptoms (63% - 127/201). Also, Biology students had the highest percentage for the utilisation of overall (17% - 35/201) and prescribed (13% - 27/201) medication for GORD symptoms. Presence of symptoms related to GORD was significantly associated with female sex [OR - 0.436 (95% CI 0.342–0.555)], being boarded [OR - 2.021 (95% CI 1.325–3.083)], chronic illness [OR - 2.632 (95% CI 1.439–4.813)], midnight snack [OR - 1.776 (95% CI 1.379–2.287)], frequent lack of breakfast [OR - 2.145 (95% CI 1.688, 2.725)], quick eating [OR - 1.394 (95% CI 1.091–1.780)] and inadequate sleep [OR - 2.077 (95% CI 1.624–2.655)].

**Conclusion:**

A high prevalence of symptoms related to GORD in comparison to previous literature was found among private tuition attendees. Possible reasons for the above findings were discussed.

## Background

Gastroesophageal reflux disease (GORD) is defined as *“a condition that develops when the reflux of stomach content causes troublesome symptoms and/or complications”* [1]. GORD is a chronic and a common condition worldwide [2] which could lead to benign or malignant complications [3]. It is more prevalent in Western countries (10–20%) compared to Asian countries (< 5%) [4]. Data on the prevalence of GORD in Sri Lanka are scarce. However, a GORD prevalence was reported as 28.5% among a Sri Lankan control group aged 15–60 years used for comparison against asthma patients [5].

Associated factors for GORD are broadly classified into genetic, demographic, behavioural and comorbid factors [6]. Psychological stress predisposes to GORD [7, 8] by causing barrier dysfunction of gastrointestinal mucosa and permeability defect in oesophageal stratified epithelia [7]. Also, spicy food, carbonated soft drinks, coffee, tea and irregular dietary habits were found associated with GORD symptoms [9–14]. Moreover, inadequate sleep was associated with GORD symptoms [9, 11, 15]. Heartburn is the most common symptom of GORD [3]. However, dysphagia, painful swallowing, haematemesis and weight loss associated with GORD should alert the physician towards a malignant pathology [16].

The frequency of GORD symptoms and quality of life have an inverse relationship [6]. However, patient’s age, number and type of symptoms were found to influence treatment-seeking behaviour [17]. Management of GORD includes lifestyle measures, pharmacological treatment and surgical interventions. Medical therapy in the management of GORD involves acid suppression with antacids, histamine-receptor blockers and proton-pump inhibitors [18]. Self-medication of antacids [19], histamine receptor blockers [20] and proton pump inhibitors [21] via over-the-counter is well practised among patients with GORD symptoms.

High school students were found susceptible to GORD [22]. In Sri Lanka, the general certificate of education (advanced level) examination is the barrier examination to enter the state-owned university. Education is free of charge for Sri Lankan students at state-owned universities. Also, students receive Mahapola scholarship, bursary and endowed scholarships via the University Grants Commission according to the merit and need [23]. The above reasons have created enormous competition among the advanced level (AL) students who have ended up having busy schedule at school and private-tuition classes. The above leads to constant psychological stress and the students would experience inadequate sleep and lack of physical exercise. A Sri Lankan study found a high prevalence of anxiety and depression among students facing barrier examinations [24]. Barriers to healthy dietary choices were also found among Sri Lankan school children [25]. Moreover, academic and psychological stress are found to be associated with alcohol consumption [26] and cigarette smoking [27]. Considering the above facts, Sri Lankan AL students could have potential risk factors for GORD symptoms. Therefore, we aimed to determine the prevalence, associated factors and medication for symptoms related to GORD among final year, AL, private-tuition attendees of the Anuradhapura municipal council area.

## Methods

### Study design and setting

A descriptive cross-sectional study was conducted in the municipal council area of Anuradhapura district among final year, AL, private-tuition attendees who were planning to sit for their AL examination in the year 2019. Anuradhapura is a rural [28], agrarian [29] district which is the largest by surface area in Sri Lanka. In 2017, around 11,200 students sat for the AL examination in Anuradhapura district out of which nearly 8700 were school candidates, and the rest were private candidates [30]. AL students of Anuradhapura (both school and private candidates) attend numerous private-tuition classes to enhance their chance of entering the state-owned universities. Private-tuition includes individual, group and mass classes. Most of the private-tuition classes are registered at the municipal council of Anuradhapura. Students sit for the AL examination in Bio-science, Physical-science, Commerce, Arts and Technology streams. Biology, Combined Mathematics, Accounts, Sinhala language and Science for Technology classes respectively are unique for each of the streams mentioned above [30]. The dietary practices in the study setting is mainly rice based. However, barriers to healthy dietary choices are found among Sri Lankan school children [25]. Due to the busy schedule, students reject traditional foods for less nutritious ‘faster’ foods [25].

### Sample size

The minimum sample size was calculated as 384 using the equation of *n* = [Z^2^xP(1-P)]/d^2^. Where n is sample size, Z is Z statistic for a level of confidence (1.96), P is expected prevalence or proportion (0.5), and d is precision (0.05) [31]. With an addition of 10% of the minimum sample size, a minimum of 425 students were needed to be recruited.

### Sampling method and selection criteria

Students sitting for AL examination in Bio-science, Physical-science, Commerce, Arts, and Technology streams were recruited from the Biology, Combined Mathematics, Accounts, Sinhala language and Science for Technology classes respectively. Registered, Sinhala medium, private-tuition classes which had the highest number of students on role for the selected AL subjects were chosen from the municipal council area of Anuradhapura district. All students aged ≥18 years who attended the selected classes were recruited.

### Study instrument

A self-administered questionnaire was used to collect data from the selected subjects. The questionnaire comprised information on demographic data, symptoms, associated factors and medication for GORD. Demographic data included the date of birth, the stream of AL examination, sex, religion, district, monthly family income, being boarded, number of attempts at the AL examination, previous illnesses and surgeries. For the household income, mean monthly household income of Anuradhapura district (58,000 Sri Lankan rupees) was considered [32]. The 12-item Frequency Scale for Symptoms of GORD by Kusano M et al. (2004) was used to collect data on symptoms related to GORD [33]. Each item was allocated to a scale of never (score = 0), occasionally (score = 1), sometimes (score = 2), often (score = 3) and always (score = 4) [33]. Seven questions were on acid reflux symptoms and five on dysmotility symptoms [34]. Individuals scoring ≥ eight on the scale were categorised to have symptoms related to GORD [33]. Questions on lifestyles related to GORD by Yamamichi N et al. (2012) were used to collect data on associated factors for symptoms related to GORD [9]. Prior permission to use the above scale and questions were obtained from the relevant principal authors. Also, following questions were asked on drugs received for GORD symptoms: name of the medications used for the GORD symptoms during the past 4 weeks, relief of symptoms following the use of the drugs, whether the drug was prescribed by a doctor, and if not, from where the drug and drug information were obtained. The subjects received the questionnaire in Sinhala language as they were recruited from Sinhala medium classes. The questionnaire was back-translated from Sinhala to English by a bilingual (Sinhala and English), English language (advanced level) teacher. The face validity of the questionnaire was established by experts and subsequently, it was pre-tested in 30 AL students to improve its content, language and sequence. The Cronbach’s alpha of questions representing acid reflux symptoms and dysmotility symptoms were 0.78 and 0.73 respectively, indicating good internal consistency in the responses [35].

### Data collection, description and analysis

Prior permissions for the data collection was obtained from relevant directors of the selected private-tuition classes. Explaining the study, obtaining verbal consent and data collection was done by the first six authors. Verbal consent following an explanation of the study was considered suitable as the study did not collect any sensitive data neither did it involve any anthropometric measurements or venipuncture and also the study subjects were ≥ 18 years of age. Data were entered into a Microsoft Excel sheet for analysis (Additional file 1). Descriptive statistics were used to describe the data. Students scoring ≥ eight on the Frequency Scale for Symptoms of GORD were categorised to have symptoms related to GORD [33]. The total score received by each GORD symptom was calculated among the participants. The study subjects were grouped into two (those who have and those who do not have symptoms related to GORD) according to the GORD score. Also, chi-square test was performed to determine the significant difference of the presence of symptoms related to GORD between the students of different AL subjects (*P* < 0.05). Pearson (95% confidence interval) correlation and regression analysis (*P* < 0.05) were performed for the GORD scores [33] against the age of the participants. Logistic regression was performed to determine the significant association between variables of interest and the presence of symptoms related to GORD (*P* < 0.05). Variables of interests were sex, religion, district, monthly household income, being boarded, number of attempts at the AL examination, presence of chronic Illness, previous surgery, exercise, midnight snack, inadequate sleep, lack of breakfast, the timing of dinner before bed, quick eating, cigarette smoking and alcohol consumption. Odds ratios with 95% confidence interval were presented for each variable of interest.

## Results

### Demographic data

Out of the 1166 AL students who participated in the study, 52 were omitted from analysis due to missing data. Therefore, 1114 questionnaires were included for final analysis (96% - 1114/1166). Science for Technology class had the highest number of students (32% - 361/1114) followed by Sinhala language (25% - 280/1114), Biology (18% - 201/1114), Combined Mathematics (13% - 147/1114) and Accounts (11% - 125/1114). The mean age of the participants was 19 (SD 1) years with a range of 18 to 27 years. Most of the study participants were females (56% - 626/1114), Buddhists (99% - 1107/1114), from Anuradhapura (90% - 998/1114), not boarded (90% - 1007/1114), about to face their first attempt at the AL examination (64% - 715/1114) and with a monthly income of < 58,000 Sri Lankan rupees (55% - 617/1114). Students were boarded (*n* = 107) at a mean of 6.5 (SD 1.13) days per week. Students about to face their second and third attempt at the AL examination were 29% (327/1114) and 7% (72/1114) respectively. Among the students, 5% (56/1114) had chronic illnesses, and out of which 45% (25/56) had a history of asthma or wheezing. Among those who had previous surgery (5% - 50/1114), 24% (12/50) have had an appendicectomy (Additional file 1).

### Prevalence of symptoms related to GORD

**Fig. 1:**
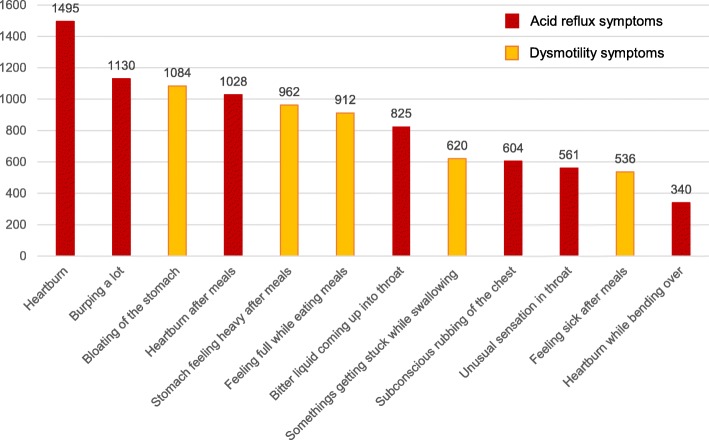
Scores received by each GORD symptom among the study participants

According to the frequency scale for symptoms of GORD, 52% (580/1114) had symptoms related to GORD (score ≥ 8). The mean score of 9.1 (SD 6.30) was observed with a range of 0 to 36. Heartburn received the highest total score of 1495 among GORD symptoms (Fig. 1). Students of the Biology class had the highest prevalence of GORD symptoms (63% - 127/201) followed by Combined Mathematics (58% - 85/147), Accounts (51% - 64/125), Sinhala language (50% - 140/280) and Science for Technology (45% - 164/361). A significant difference in the presence of symptoms related to GORD was found between the students of different AL subjects (*P* = 0.0009). Mean score for symptoms of gastroesophageal reflux disease by AL subjects is summarised in Table 1.

**Table 1:** Mean score for symptoms of gastroesophageal reflux disease by subject

Questions	Overall (***n*** = 1114)		Subjects									
			Biology (***n*** = 201)		Combined Mathematics (***n*** = 147)		Accounts (***n*** = 125)		Sinhala (***n*** = 280)		Science for Technology (***n*** = 361)	
	Mean	SD	Mean	SD	Mean	SD	Mean	SD	Mean	SD	Mean	SD
***Acid reflux symptoms***												
1. Do you get heartburn?	1.34	0.84	1.52	0.85	1.39	0.95	1.38	0.91	1.32	0.77	1.22	0.80
2. Do you sometimes subconsciously rub your chest with your hand?	0.54	0.84	0.58	0.82	0.62	0.95	0.63	0.91	0.47	0.77	0.52	0.84
3. Do you get heartburn after meals?	0.92	0.93	1.15	0.89	0.88	0.92	0.94	0.90	0.95	1.00	0.79	0.88
4. Do you have an unusual (e.g. burning) sensation in your throat?	0.50	0.75	0.58	0.84	0.48	0.75	0.46	0.72	0.50	0.78	0.49	0.67
5. Do you get bitter liquid (acid) coming up into your throat?	0.74	0.91	0.94	0.98	0.86	0.94	0.75	0.95	0.65	0.91	0.65	0.84
6. Do you burp a lot?	1.01	0.96	1.23	0.89	1.18	1.10	0.98	0.95	0.93	0.98	0.91	0.91
7. Do you get heartburn if you bend over?	0.31	0.64	0.32	0.62	0.33	0.66	0.27	0.59	0.31	0.68	0.29	0.63
***Dysmotility symptoms***												
8. Does your stomach get bloated?	0.97	0.85	1.16	0.85	0.95	0.82	0.92	0.89	0.93	0.87	0.93	0.84
9. Does your stomach ever feel heavy after meals?	0.86	0.96	1.10	1.05	0.87	0.97	0.74	0.87	0.86	0.97	0.77	0.91
10. Do you ever feel sick after meals?	0.48	0.76	0.78	0.86	0.48	0.77	0.43	0.70	0.40	0.76	0.40	0.69
11. Do you feel full while eating meals?	0.82	0.96	0.92	0.88	0.87	1.11	0.78	0.89	0.81	1.01	0.76	0.93
12. Do some things get stuck when you swallow?	0.56	0.80	0.56	0.77	0.62	0.85	0.61	0.78	0.55	0.83	0.52	0.77

### Associated factors for symptoms related to GORD

The percentage for the presence of associated factors for gastroesophageal reflux disease by AL subjects are summarised in Table 2. There was no correlation between the age of the participants and the GORD symptom scores (r = 0.056, *P* = 0.06). Sixty-one per cent (382/626) of females had GORD symptoms (score ≥ 8), in comparison to 41% (198/488) of males. Sixty-seven per cent (72/107) of students who were boarded had GORD symptoms, in comparison to 51% (508/1007) of who were not boarded. Seventy-three per cent (41/56) of students who had a chronic illness was having GORD symptoms, in comparison to 50% (516/1035) of who had no chronic illnesses. Sixty-one per cent (231/376) of students who had a habit of midnight snack for more than three times a week were having GORD symptoms, in comparison to 47% (349/738) of who did not have the habit. Sixty-one per cent (345/562) of students who had frequent lack of breakfast for more than three times a week were having GORD symptoms, in comparison to 43% (235/552) of who did not have the habit. Fifty-seven per cent (237/414) of students who had a habit of quick eating was having GORD symptoms, in comparison to 49% (343/700) of who did not have a habit of quick eating. Sixty-three per cent (278/442) of students who had inadequate sleep were having GORD symptoms, in comparison to 45% (302/672) of who had an adequate sleep.

**Table 2:** Associated factors for gastroesophageal reflux disease by subject

Questions	Overall (***n*** = 1114)		Subjects									
			Biology (***n*** = 201)		Combined Mathematics (***n*** = 147)		Accounts (***n*** = 125)		Sinhala (***n*** = 280)		Science for Technology (***n*** = 361)	
	Yes	No	Yes	No	Yes	No	Yes	No	Yes	No	Yes	No
1. Is your time of exercise less than 30 min a day?	79%	21%	86%	14%	83%	17%	77%	23%	76%	24%	76%	24%
2. Do you have a habit of midnight snack (more than three times a week)?	34%	66%	40%	60%	33%	67%	26%	74%	31%	69%	35%	65%
3. Do you have a feeling of inadequate sleep?	40%	60%	52%	48%	51%	49%	32%	68%	34%	66%	35%	65%
4. Do you have a habit of frequent lack of breakfast (more than three times a week)?	50%	50%	51%	49%	44%	56%	53%	47%	55%	45%	48%	52%
5. Do you have a habit of having dinner within two hours before going to bed?	45%	55%	37%	63%	44%	56%	49%	51%	46%	54%	47%	53%
6. Do have a habit of quick eating?	37%	63%	40%	60%	45%	55%	34%	66%	26%	74%	42%	58%
7. Do you have a habit of smoking?	3%	97%	2%	98%	5%	95%	2%	98%	1%	99%	6%	94%
8. Do have a habit of alcohol drinking (almost every day)?	2%	98%	2%	98%	1%	99%	2%	98%	1%	99%	4%	96%

Logistic regression revealed that female sex [OR - 0.436 (95% CI 0.342–0.555)], being boarded [OR - 2.021 (95% CI 1.325–3.083)], chronic illness [OR - 2.632 (95% CI 1.439–4.813)], midnight snack [OR - 1.776 (95% CI 1.379–2.287)], frequent lack of breakfast [OR - 2.145 (95% CI 1.688, 2.725)], quick eating [OR - 1.394 (95% CI 1.091–1.780)] and inadequate sleep [OR - 2.077 (95% Ci 1.624–2.655)] were significantly associated with GORD symptoms. However, there was no significant association between the presence of symptoms related to GORD and the following variables of interest: religion, district, monthly household income, number of attempts at the AL examination, previous surgery, exercise, the timing of dinner before bed, cigarette smoking and alcohol consumption (Table 3).

**Table 3:** Presence of GORD symptoms against the variables of interest

Item	Description	Presence of GORD	Absence of GORD	Logistic Regression		Odds ratio (95% CI)
				Co efficient	***P*** Value	
*1. Sex*	*Male*	*198*	*290*	*−0.923*	*< 0.001*	*0.436 (0.342–0.555)*
	*Female*	*382*	*244*			
2. Religion	Buddhist	576	531	−0.676	0.413	0.813 (0.181–3.65)
	Non-Buddhist	4	3			
3. District	Anuradhapura	520	478	0.130	0.561	1.015 (0.691–1.492)
	Other districts	60	56			
4. Income	≥58,000 Income	264	233	−0.027	0.838	1.079 (0.851–1.367)
	< 58,000 Income	316	301			
*5. Boarded*	*Yes*	*72*	*35*	*0.637*	*0.008*	*2.021 (1.325–3.083)*
	*No*	*508*	*499*			
6. Examination attempt	First attempt	352	363	−0.226	0.101	0.727 (0.568, 0.930)
	Other attempts	228	171			
*7. Chronic Illness*	*Presence*	*41*	*15*	*0.864*	*0.008*	*2.632 (1.439–4.813)*
	*Absence*	*539*	*519*			
8. Past Surgery	Presence	26	24	−0.169	0.596	0.997 (0.565–1.759)
	Absence	554	510			
9. Is your time of exercise less than 30 min a day?	Yes	474	404	0.044	0.788	1.439 (1.078–1.921)
	No	106	130			
*10. Do you have a habit of midnight snack (more than three times a week)?*	*Yes*	*231*	*145*	*0.434*	*0.002*	*1.776 (1.379–2.287)*
	*No*	*349*	*389*			
*11. Do you have a feeling of inadequate sleep?*	*Yes*	*278*	*164*	*0.641*	*< 0.001*	*2.077 (1.624–2.655)*
	*No*	*302*	*370*			
*12. Do you have a habit of frequent lack of breakfast (more than three times a week)?*	*Yes*	*345*	*217*	*0.573*	*< 0.001*	*2.145 (1.688, 2.725)*
	*No*	*235*	*317*			
13. Do you have a habit of having dinner within two hours before going to bed?	Yes	263	236	0.157	0.228	1.048 (0.827–1.327)
	No	317	298			
*14. Do have a habit of quick eating?*	*Yes*	*237*	*177*	*0.376*	*0.006*	*1.394 (1.091–1.780)*
	*No*	*343*	*357*			
15. Do you have a habit of smoking?	Yes	22	16	0.201	0.613	1.276 (0.663–2.457)
	No	558	518			
16. Do have a habit of alcohol drinking (almost every day)?	Yes	14	10	0.193	0.700	1.296 (0.571–2.943)
	No	566	524			

Italic values indicate significance with a *p*-value of < 0.05

### Medication for GORD symptoms

**Fig. 2:**
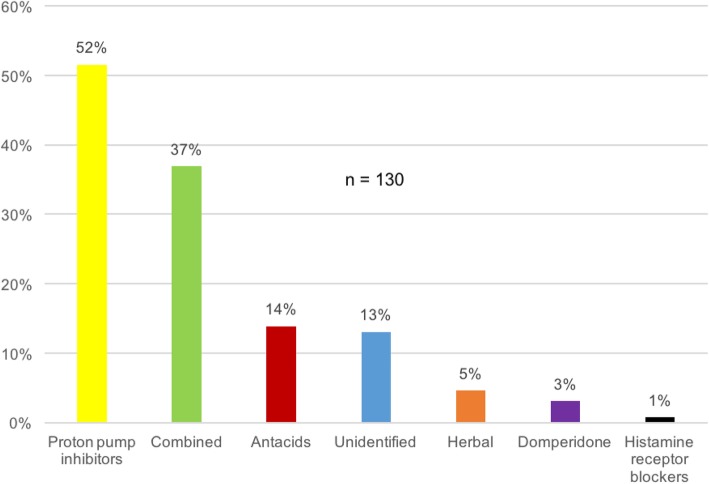
Medications used for the symptoms of gastro-oesophageal reflux disease by the study participants

Overall 130 out of 1114 students have had medications for symptoms related to GORD (12%). Symptom relief with medication was experienced by 89% (115/130). Proton pump inhibitors (52% - 67/130) were used by most of the students (Fig. 2 and Additional file 1). Thirty-five per cent of the participants (45/130) have self-medicated for GORD symptoms. Out of those who self-medicated, most have got their medication from a pharmacy (71% - 32/45) followed by household (27% - 12/45) and friends (2% - 1/45). The drug information for self-medication was obtained mostly from family (25/45) followed by prior knowledge (8/45), pharmacist (6/45), internet (5/45) and friends (1/45). Biology students had the highest percentage for the utilisation of overall (17% - 35/201) and prescribed (13% - 27/201) medication. However, students of Combined Mathematics showed the highest percentage of self-medication for GORD symptoms (7% - 11/147) (Table 4).

**Table 4:** Drug utilisation by subject

Items	Overall (***n*** = 1114)	Subjects				
		Biology (***n*** = 201)	Combined Mathematics (***n*** = 147)	Accounts (***n*** = 125)	Sinhala (***n*** = 280)	Science for Technology (***n*** = 361)
Drug utilization	130 (12%)	35 (17%)	21 (14%)	9 (7%)	22 (8%)	43 (12%)
Prescribed drug utilization	85 (8%)	27 (13%)	10 (7%)	6 (5%)	17 (6%)	25 (7%)
Self-medication	45 (4%)	8 (4%)	11 (7%)	3 (2%)	5 (2%)	18 (5%)

## Discussion

The study showed a high prevalence of 52% for symptoms related to GORD among AL, private tuition attendees in Anuradhapura municipal council. The prevalence was much higher compared to Asian [4] and local [5] data. However, the present study focused only on the symptoms related to GORD. A 25% prevalence of GORD was seen among a medical student group of India [36]. Also, inadequate sleep, missing breakfast regularly and quick eating were significantly associated with GORD [36]. Thirty-one percent and 30% of South Indian medical student group had at least one episode of heartburn and regurgitation per week respectively [13]. Moreover, heart burn was seen in 22% of medical students in Karachi, Pakistan [37]. The above facts revealed that GORD symptoms have been a significant health issue among South Asian student population.

Student being boarded was significantly associated with the presence of GORD. When a student is boarded away from home, he or she would be more prone to risk factors of GORD such as poor dietary habits and psychological stress. Our study found dietary habits like midnight snacks, frequent lack of breakfast and quick eating to be significantly associated with GORD. Quick eating is possible in a population which is currently experiencing a busy schedule due to the impending barrier examination in their life. Previous evidence suggests irregular dietary habits (midnight snack, frequent lack of breakfast, dinner just before bedtime and quick eating) to be associated with GORD symptoms [9–14]. A significant association between inadequate sleep and GORD found in the present study reinforces the findings of prior literature [9, 11, 15]. In contrary to previous findings [6, 9, 22, 34], our findings did not show a significant association for GORD with cigarette smoking and alcohol consumption. However, the prevalence of cigarette smoking and alcohol consumption among the selected population was 3% and 2% respectively. Our study also found a significant association between the presence of chronic illness and GORD. Among those who had chronic illnesses, 45% (25/56) had a history of asthma or wheezing. Asthma is one of the comorbid factors for GORD [6]. Amarasiri LD et al. 2010 found high prevalence of GORD symptoms among Sri Lankan adult asthmatics and stated that asthma could influence the presence of GORD symptoms [5].

Findings of a cross-sectional study conducted amongst a focused group of a particular municipal council cannot establish a causal association and neither it could be generalised. A large-scale survey among private tuition attendees would be ideal to further emphasise the newly found high prevalence of GORD among private tuition attendees. However, present data are unique as it was from a fairly large population (*n* = 1114) in a rural region of Sri Lanka. Moreover, associated factors for GORD such as poor dietary habits and inadequate sleep need to be addressed among the target population as GORD symptoms could hinder the student performance at examinations.

## Conclusion

A high prevalence of GORD was found among private tuition attendees along with a significant association for certain demographic, dietary and behavioural factors. Addressing such factors could help minimise the burden of GORD and subsequently enhance the performance of students at examinations.

## Supplementary information

**Additional file 1.** Prevalence, associated factors and medication for GORD among private tuition attendees of Anuradhapura, Sri Lanka – 2019. Description of data –This contains the data of the entire study

## Data Availability

All data generated or analysed during this study are included in this published article (and its additional files).

## Abbreviations

AL: Advance level
GORD: Gastro-oesophageal reflux disease
